# Complete pathological response after neoadjuvant chemoimmunotherapy in PD-L1-negative unresectable primary hepatic small cell carcinoma: a case report and tumor microenvironment analysis

**DOI:** 10.3389/fimmu.2026.1901608

**Published:** 2026-09-11

**Authors:** Yupeng Hong, Sifu Yang, Yuchen Zhong, Yuan Chen, Liu Yang

**Affiliations:** 1 Cancer Center, Department of Medical Oncology, Zhejiang Provincial People’s Hospital, Affiliated People’s Hospital, Hangzhou Medical College, Hangzhou, Zhejiang, China; 2 Department of Pathology, Zhejiang Provincial People’s Hospital, Affiliated People’s Hospital, Hangzhou Medical College, Hangzhou, Zhejiang, China

**Keywords:** complete pathological response, neoadjuvant chemoimmunotherapy, PD-L1 negative, primary hepatic small cell carcinoma, tumor microenvironment

## Abstract

**Background:**

Primary hepatic small cell carcinoma (PHSCC) is an extraordinarily rare and aggressive malignancy with a dismal prognosis. No standard treatment exists, and the role of immunotherapy in this tumor type, especially in programmed cell death ligand 1 (PD-L1) negative cases, remains unexplored.

**Case presentation:**

A 55-year-old woman presented with a large, unresectable liver mass. Biopsy confirmed a neuroendocrine carcinoma, small cell type, with a PD-L1 Combined Positive Score (CPS) of 0. She received neoadjuvant therapy with cisplatin/carboplatin, etoposide, and serplulimab; after the first cycle, cisplatin was switched to carboplatin due to renal impairment. After 4 cycles, imaging showed a partial response, allowing for successful surgical resection. Pathological examination of the resected specimen revealed a complete pathological response (pCR) with no viable tumor cells. Multiplex immunofluorescence (mIF) analysis of the tumor microenvironment post-resection revealed an immune-rich stroma, characterized by a high density of CD38+ cells (800 cells/mm^2^) and PD-L1+ macrophages, alongside a prominent intratumoral CD8+ T-cell infiltrate.

**Conclusion:**

This case demonstrates that neoadjuvant chemoimmunotherapy can achieve pCR and facilitate surgical resection in initially unresectable PHSCC, even with a negative PD-L1 status. The response may be mediated by a chemotherapy-synergized, PD-1 inhibitor-driven immune activation within a receptive tumor microenvironment, particularly the stromal compartment. The presence of an immune-rich stroma may serve as a critical determinant for immunotherapy efficacy, highlighting the limitations of relying solely on tumor cell PD-L1 expression as a biomarker in this rare malignancy.

## Introduction

Primary hepatic small cell carcinoma (PHSCC) is a malignancy of extreme rarity, with its diagnosis requiring the exclusion of a more common small cell carcinoma primary from another site, typically the lung (1–5). Its histopathological and immunophenotypic features mirror those of small cell lung cancer (SCLC), characterized by small, hyperchromatic cells with scant cytoplasm and expression of neuroendocrine markers such as chromogranin A, synaptophysin, and CD56 (6). Due to its aggressive biology and propensity for early metastasis, the prognosis for PHSCC is universally poor, with limited long-term survival data available from the sparse case reports in the literature (7, 8). The absence of standardized treatment guidelines means management is often empirically derived from SCLC protocols, which have traditionally relied on platinum-etoposide chemotherapy (9, 10).

The advent of immune checkpoint inhibitors (ICIs) has transformed the therapeutic landscape for several malignancies, including a subset of SCLC (11). However, PD-L1 expression has shown variable predictive value for ICI response in SCLC, and its significance in PHSCC is entirely unknown (12, 13). Responses to ICIs in PD-L1 negative SCLC patients have been documented, suggesting that other factors within the complex ecosystem of the tumor microenvironment (TME) are pivotal (14, 15).

We present a compelling rare case of a patient with initially unresectable, PD-L1 negative (CPS = 0) PHSCC who achieved a complete pathological response (pCR) following neoadjuvant therapy with carboplatin, etoposide, and the PD-1 inhibitor Serplulimab. This report not only describes a successful multidisciplinary approach to a formidable clinical challenge but also utilizes post-treatment multiplex immunofluorescence to interrogate the TME. We hypothesize that the robust clinical response, despite negative PD-L1 expression, was facilitated by a synergistic interaction between chemotherapy and immunotherapy within an immune-reactive stromal niche, offering novel insights into the mechanism of action in this rare entity.

## Case presentation

**Figure 1:**
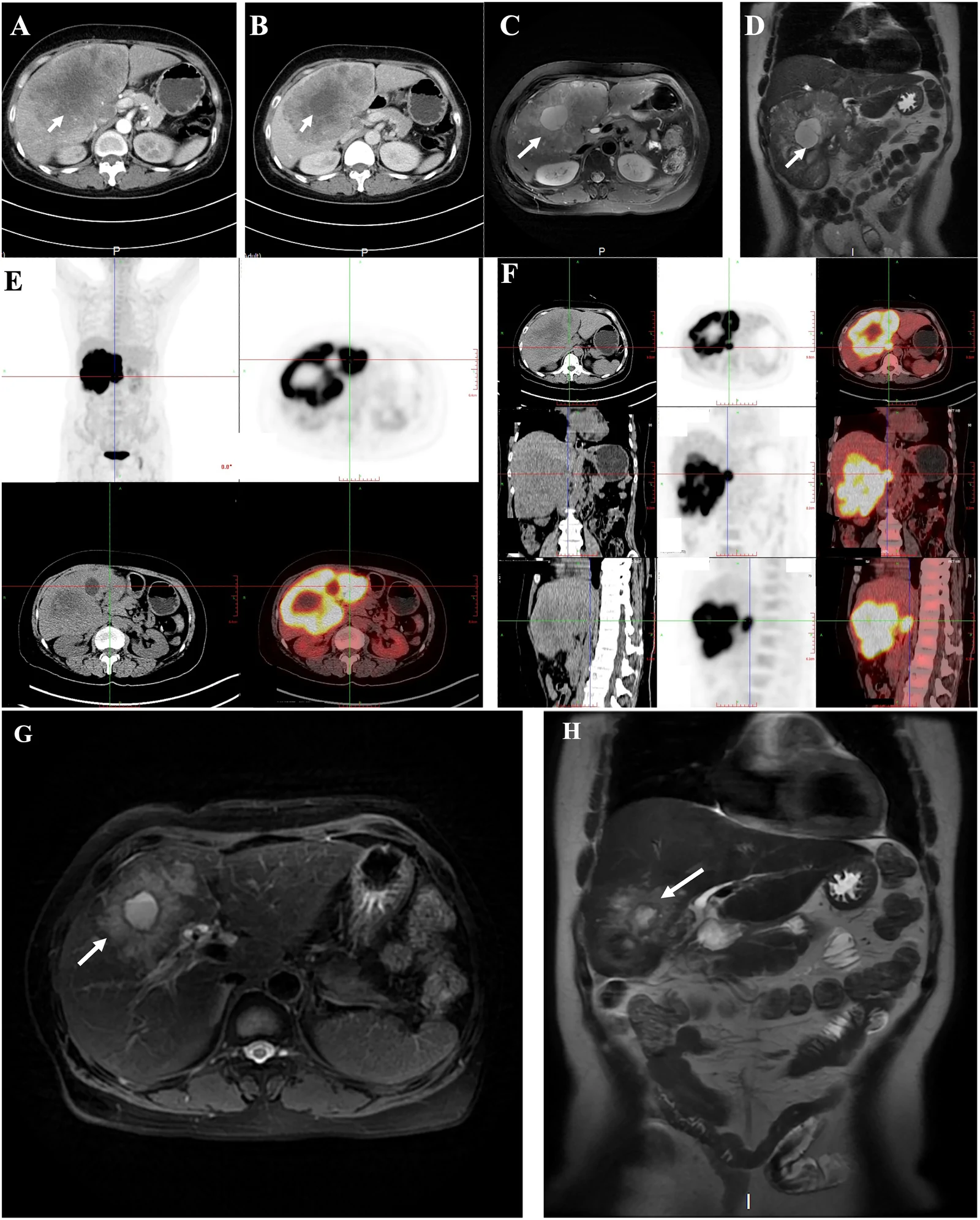
Diagnostic Imaging at Initial Presentation. **(A)** Arterial-phase of Contrast-Enhanced Abdominal CT image demonstrated intense heterogeneous enhancement of the large (14.5 × 9.2 × 13.6 cm) lobulated mass in the right hepatic lobe; **(B)** Venous-phase of image Contrast-Enhanced Abdominal CT image showed washout with persistent peripheral enhancement and internal non-enhancing necrotic areas; **(C)** T2-weighted of Contrast-Enhanced MRI image showed a large lobulated mass (13.1 × 10.0 × 13.6 cm) in the right hepatic lobe with heterogeneous hyperintensity and peripheral satellite nodules; **(D)** Coronal T2-weighted image of Contrast-Enhanced MRI demonstrated mass effect on intrahepatic vasculature and irregular gallbladder wall thickening; **(E, F)** PET-CT image demonstrates intense, heterogeneous FDG uptake in the right hepatic lobe (SUV_max_=21.4) with central necrosis, and a separate focus of increased uptake in the porta hepatis (SUVmax=18.0). Axial, Coronal and Sagittal PET/CT image revealed the large (15.0 × 9.4 × 14.8 cm), hypodense hepatic mass with peripheral FDG avidity and central necrosis. **(G, H)** Post-chemotherapy MRI. Irregular lobulated mass in right lobe measuring 8.2 × 5.7 × 5.6 cm. White arrows indicated the mass.

A 55-year-old female with no significant past medical history presented to our hospital with complaints of abdominal pain accompanied by nausea and vomiting. Physical examination was unremarkable. Laboratory investigations revealed a significantly elevated level of neuron-specific enolase (NSE) (102.0 ng/ml, upper limit of normal=20 ng/ml). Routine blood tests, liver function tests, and other tumor markers were within normal ranges. Contrast-enhanced computed tomography of the abdomen revealed a large, lobulated, hypodense mass measuring approximately 145×92×136 mm in the right lobe of the liver, demonstrating vivid enhancement in the arterial phase with washout in the venous phase. A smaller satellite nodule was also noted. These findings were highly suspicious for a primary malignant liver tumor, such as massive hepatocellular carcinoma with a daughter nodule. Subsequent magnetic resonance imaging (MRI) corroborated the CT findings, providing better soft tissue characterization (**Figures 1A, B, C, D**). Chest CT showed no pulmonary mass, nodules, or suspicious mediastinal lymphadenopathy. For further staging and characterization, a positron emission tomography-computed tomography (PET-CT) scan was performed, which demonstrated heterogeneous FDG avidity within the large liver mass and the adjacent satellite nodule, confirming a hypermetabolic primary hepatic malignancy with no evidence of distant metastases (**Figures 1E, F**).

**Figure 2:**
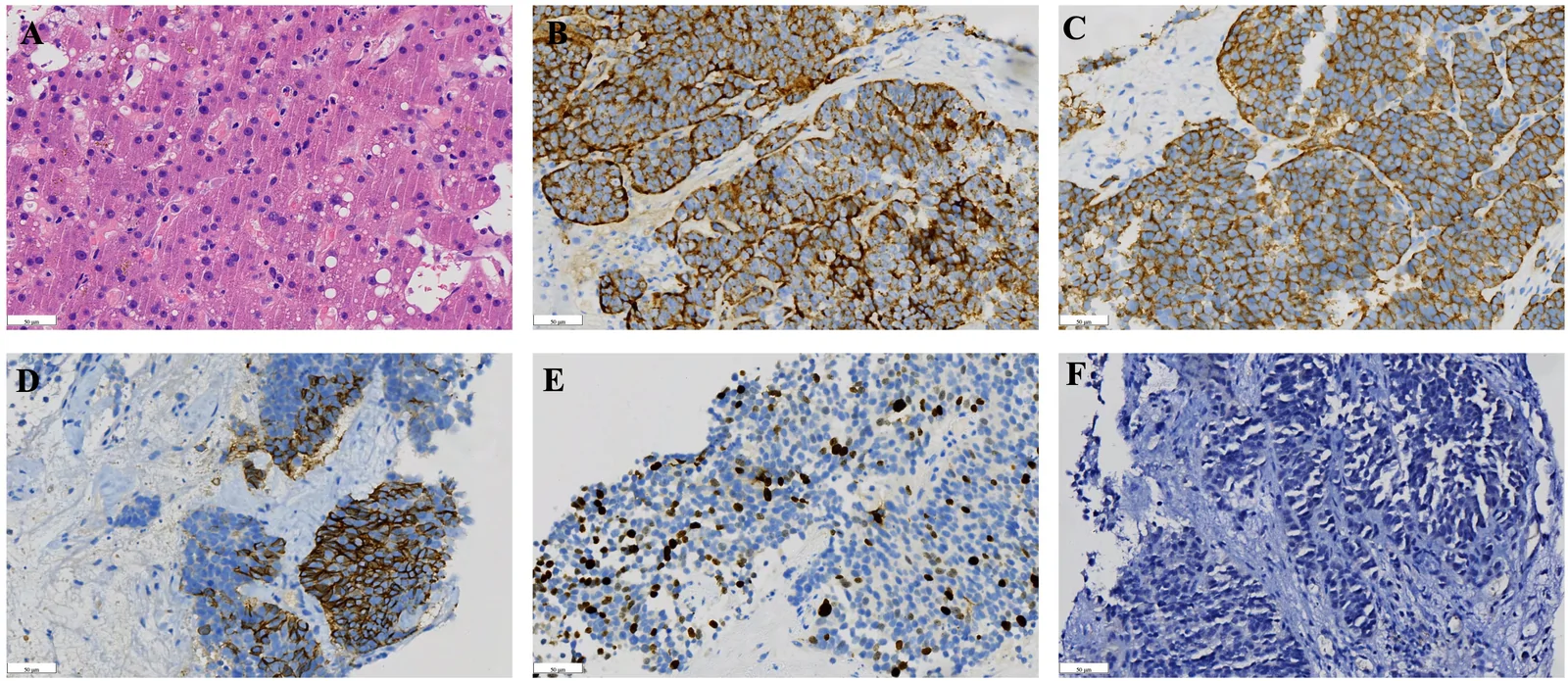
Immunohistochemical staining profile of the liver biopsy specimen(×200). **(A)** Histopathology of small cell carcinoma; **(B–D)** Positive staining for neuroendocrine markers: Chromogranin A (CgA+), Synaptophysin (SYN+), and focal CD56+ respectively; **(E)** Ki67 proliferation index: 20%; **(F)** PD-L1 (22C3) testing: Combined Positive Score (CPS) = 0 (negative).Scale bars:50μm.

An ultrasound-guided core needle biopsy of the liver mass was performed. Histopathological examination revealed a malignant neoplasm composed of small, hyperchromatic cells with scant cytoplasm, consistent with a small cell carcinoma. Immunohistochemistry (IHC) was positive for chromogranin A (CgA), synaptophysin (SYN), and CD56 (focal), supporting a diagnosis of neuroendocrine carcinoma, small cell type. The Ki-67 proliferation index was approximately 20%. PD-L1 testing using the 22C3 pharmDx assay yielded a Combined Positive Score (CPS) of 0 (**Figure 2**).

After a multidisciplinary tumor board review, the patient was deemed not to be a candidate for upfront surgery due to the large size of the tumor and the presence of a satellite lesion. It was decided to initiate neoadjuvant systemic therapy. The patient commenced treatment with cisplatin, etoposide, and serplulimab (a PD-1 inhibitor). Serplulimab, an anti-PD-1 antibody, has shown survival benefit in extensive-stage SCLC in the ASTRUM-005 trial. Given the histologic and immunophenotypic similarity between PHSCC and SCLC, we applied this regimen in a neoadjuvant setting (16). This decision also considered drug accessibility in our institution and the absence of standard therapy for PHSCC. Because serplulimab is not approved for primary hepatic small cell carcinoma, its off-label use was discussed with the patient, and written informed consent was obtained. After the first cycle, cisplatin was switched to carboplatin due to the development of renal impairment. The patient tolerated the subsequent cycles of carboplatin/etoposide/serplulimab well. Although the change from cisplatin to carboplatin could theoretically influence response, both are standard platinum agents in small cell carcinoma, and the patient ultimately received four cycles of platinum-etoposide plus serplulimab. After 4 cycles of therapy, a follow-up MRI was performed, which showed a remarkable reduction in the size of the dominant mass and the complete resolution of the satellite nodule, meeting the RECIST 1.1 criteria for a partial response (PR) (**Figures 1G, H**). Re-evaluation by the multidisciplinary team concluded that the disease had been successfully downstaged, and the patient was now a suitable candidate for surgical resection. The patient underwent an uncomplicated laparoscopic liver resection.

**Figure 3:**
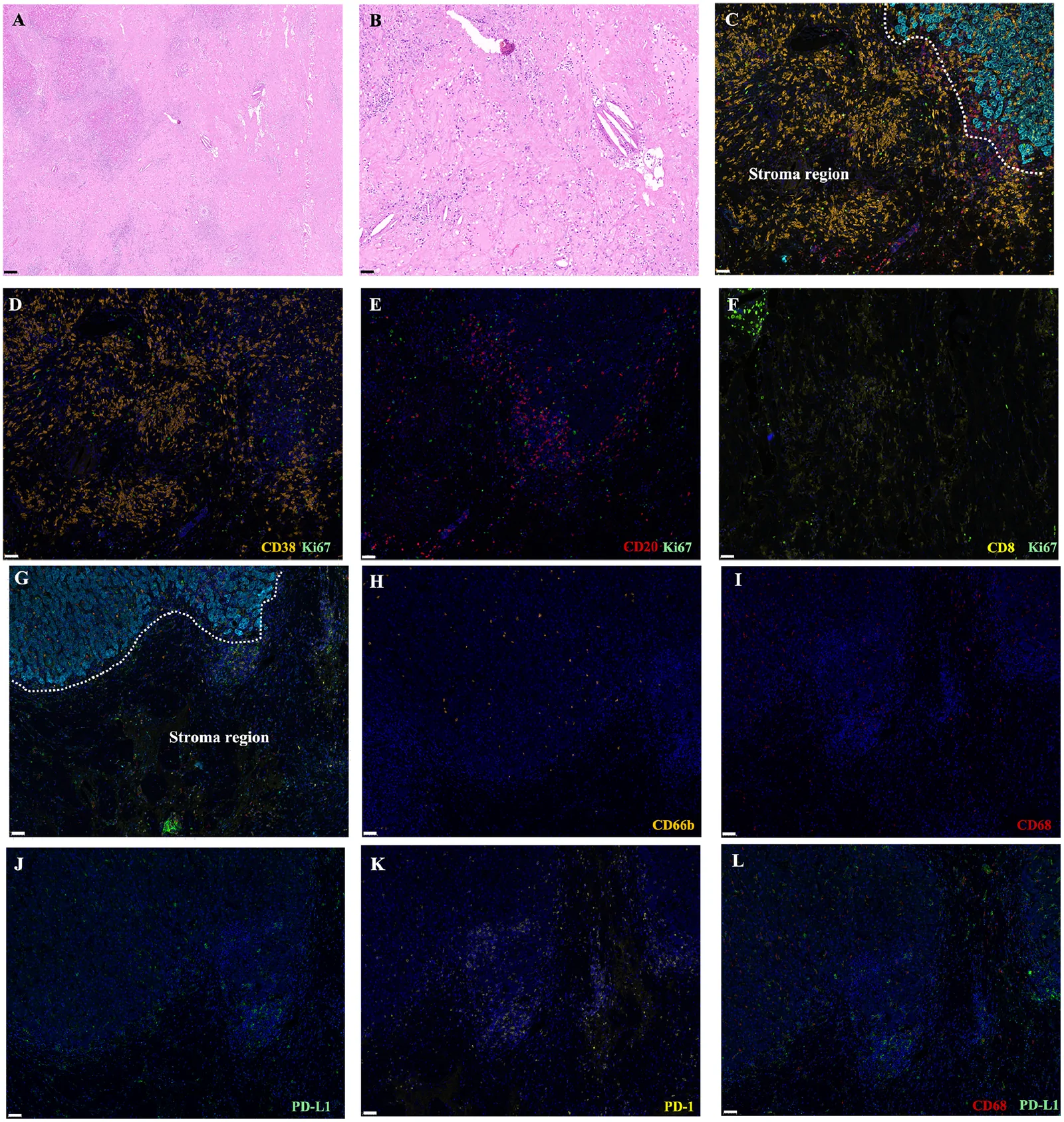
HE and mIF staining. **(A)** HE illustration of tumor tissue (magnification×50) **(B)** HE illustration of tumor tissue (magnification×200) **(C)** mIF of postoperative pathology **(A)** CD8, CD20, CD38, Ki67, CK, DAPI **(D–F)** Specific immunofluorescence channel of tumor region and stroma region. The markers of mIF are DAPI (blue), CD38(orange), CD20(red), Ki67(green), CD8(yellow), CK (bright blue); **(G)** mIF of postoperative pathology Panel **(B)** CD66b, PD-L1, CD68, PD-1, CK, DAPI. **(H–L)** Specific immunofluorescence channel of tumor region and stroma region. The markers of mIF are DAPI (blue), CD66b(orange), CD68(red), PD-L1(green), PD-1(yellow), CK (bright blue); CK, cytokeratin; DAPI, 4’,6-diamidino-2-phenylindole; HE, hematoxylin and eosin; mIF, multiplex immunofluorescence: PD-1, programmed death receptor 1; PD-L1, expressing programmed cell death 1 ligand 1. Scale bars: **(A)** 200μm. **(B–L)** 60μm.

The resected specimen was subjected to thorough histopathological examination. Macroscopically, the tumor bed appeared fibrotic and necrotic. Microscopically, the sections showed extensive areas of necrosis, fibrosis, hyalinization, and a robust inflammatory infiltrate comprising lymphocytes, plasma cells, histiocytes (foam cells), and multinucleated giant cells. Scattered microabscesses were also noted. Critically, after extensive sampling and examination, no viable tumor cells were identified (**Figures 3A, B**). The pathological findings were consistent with a complete pathological response (pCR) to neoadjuvant therapy.

To further characterize the tumor immune microenvironment, multiplex immunofluorescence (mIF) analysis was performed on the surgically resected specimen using Panel A(4’,6-diamidino-2-phenylindole (DAPI), pan-cytokeratin (pan-CK), CD8, CD20,CD38,Ki67) and Panel B(CD66b,CD68,PD-L1,PD-1,CK,DAPI)(**Figures 3C–L**, **Supplementary Materials**). The results revealed distinct spatial distributions of immune cell subsets within the tumor and stromal compartments (**Table 1**). Cytotoxic CD8+ T cells were present at a higher density in the tumor area (280 cells/mm^2^) compared to the stroma (135 cells/mm^2^). In contrast, CD38+ cells were more abundant in the stroma (800 cells/mm^2^) than in the tumor region (200 cells/mm^2^). B cells (CD20+) were sparsely distributed in the tumor area (4.5 cells/mm^2^) but were more frequent in the stroma (238 cells/mm^2^). Neutrophils (CD66b+) and macrophages (CD68+) were observed in both compartments, with macrophages being notably abundant. Furthermore, PD-L1+ macrophages (CD68+PD-L1+) were identified in both the tumor (73 cells/mm^2^) and stromal regions (196 cells/mm^2^), suggesting a potential role in immune modulation despite the initial PD-L1 CPS of 0 in the pre-treatment biopsy.

**Table 1:** Density and spatial distribution of immune cell subsets in the tumor microenvironment post-neoadjuvant therapy.

Cell type	Tumor area* (density of cells, per mm^2^)	Stroma area* (density of cells, per mm^2^)
CD8+cells (Cytotoxic T cell)	280	135
CD38+ cells	200	800
CD20+cells (B cell)	4.5	238
CD66b+cells(neutrophil)	21	91
CD68+cells (Macrophage)	195	392
CD68+PDL1+cells (PD-L1+Macrophage)	73	196

*Data were obtained from the only available post-treatment resection specimen; Multiple regions of interest (ROIs) were analyzed to minimize sampling bias. Cell densities were calculated as the average number of positive cells per mm^2^ across the selected ROIs using QuPath v0.4.3 (University of Edinburgh, UK). Because only one resection specimen was available, the analysis is descriptive and should be interpreted with caution.

**Figure 4:**
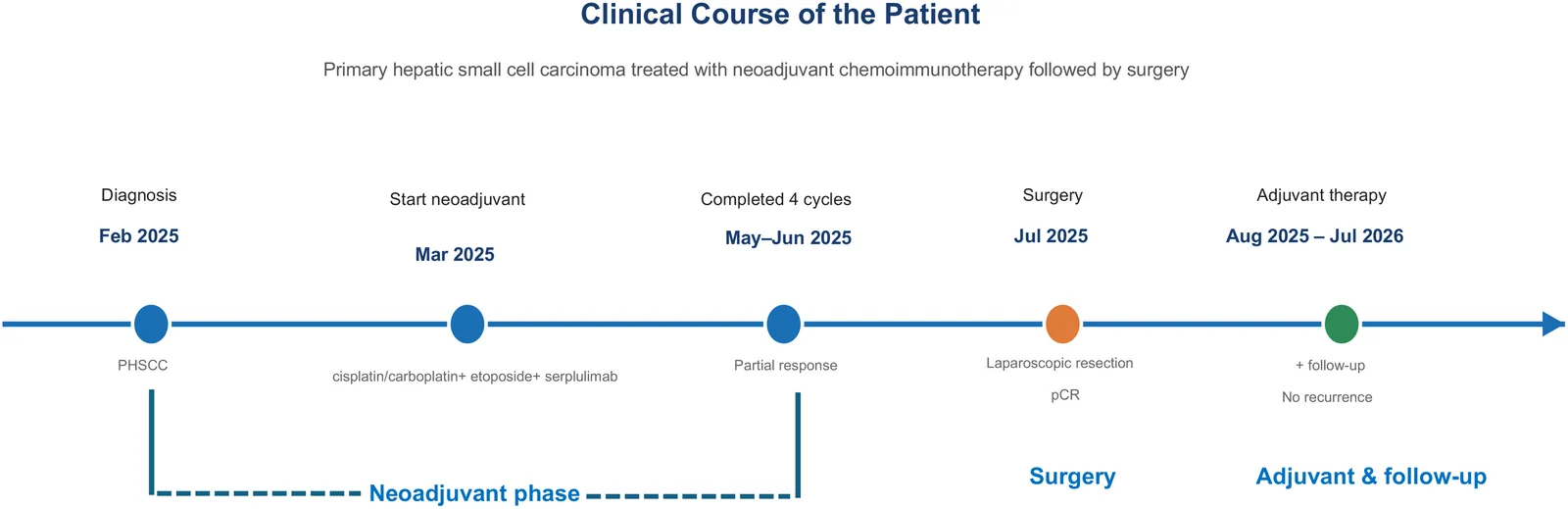
Clinical course of the patient. Timeline summarizing the key events from diagnosis to the most recent follow-up. The patient was diagnosed with primary hepatic small cell carcinoma (PHSCC) in February 2025 and received neoadjuvant chemoimmunotherapy with cisplatin/carboplatin, etoposide, and serplulimab from March to June 2025. After completing four cycles with a partial response, she underwent laparoscopic liver resection in July 2025, and pathological examination confirmed a complete pathological response (pCR). She subsequently received two cycles of adjuvant chemoimmunotherapy and remained without evidence of recurrence at the latest follow-up in July 2026.

Following surgery, the patient received 2 additional cycles of adjuvant chemoimmunotherapy(carboplatin/etoposide/serplulimab). She declined further maintenance immunotherapy. The patient continued regular surveillance with imaging every 3 months. Moreover, serum neuron-specific enolase (NSE) was monitored longitudinally. At baseline, NSE was elevated at 102.0 ng/ml. After initiation of chemoimmunotherapy, NSE declined substantially and had normalized to 12.4 ng/ml. During subsequent follow-up, NSE remained within the normal range (10.5–15.0 ng/ml), with the most recent value of 15.0 ng/ml in July 2026. At the latest follow-up, 12 months after surgery, she remains alive with no evidence of disease recurrence (**Figure 4**).

All procedures performed in this study were in accordance with the ethical standards of the institutional and/or national research committee(s) and with the Helsinki Declaration. Written informed consent was obtained from the patient for the publication of this case report and accompanying images.

## Discussion

Primary hepatic small cell carcinoma (PHSCC) is an exceptionally rare and highly aggressive malignancy, with only a handful of cases reported in the literature to date (1). Its diagnosis is based on histopathological and immunohistochemical features identical to those of small cell carcinoma of the lung. The Ki-67 index in this case was approximately 20%, which is lower than the high proliferative index commonly seen in small cell carcinoma. This may reflect tumor heterogeneity or limited sampling by core needle biopsy. Nevertheless, the morphological features and neuroendocrine marker expression were diagnostic. This finding also suggests that PHSCC may show biological heterogeneity and that Ki-67 alone should not override morphological and immunohistochemical criteria. No genomic or transcriptomic profiling was performed, which limits our ability to define the molecular background of this rare tumor or to identify predictive biomarkers. Future studies should incorporate molecular analysis. The prognosis is universally poor, and due to its rarity, no standardized treatment guidelines exist. Management is often extrapolated from protocols for small cell lung cancer (SCLC) or other hepatic malignancies. This case is therefore significant as it documents a successful multidisciplinary approach using neoadjuvant chemoimmunotherapy followed by surgery, resulting in a complete pathological response in a patient with initially unresectable disease.

A particularly intriguing aspect of this case is the achievement of pCR following anti-PD-1 immunotherapy combined with chemotherapy, despite the pre-treatment biopsy yielding a PD-L1 Combined Positive Score (CPS) of 0. It should be noted that the PD-L1 CPS from the initial biopsy and the mIF findings from the resection specimen cannot be directly compared, as they derive from different time points and use different methods. The mIF data reflect the post-treatment microenvironment only. In many cancers, PD-L1 expression has been used as a predictive biomarker for response to immune checkpoint inhibitors (17); however, its predictive value is imperfect, and responses in PD-L1 negative patients, especially in SCLC and its variants, have been observed (18). This suggests that other mechanisms within the tumor microenvironment (TME) are critical for mediating a therapeutic response. In this rare case, serplulimab was selected based on the ASTRUM-005 trial in extensive-stage small cell lung cancer. Although its use in PHSCC is off label, the decision was made after multidisciplinary discussion and with the patient’s written informed consent. Our analysis of the resected specimen via multiplex immunofluorescence provided valuable insights into these potential mechanisms. The observed pCR is likely the result of a synergistic effect. Chemotherapy may have primed the immune response by inducing immunogenic cell death, releasing tumor neoantigens, and depleting immunosuppressive cells (19). This created a more permissive TME for the anti-PD-1 antibody (serplulimab) to unleash pre-existing anti-tumor immunity.

Crucially, our mIF findings illuminate the potential mechanism behind this efficacy. The high density of CD8+ cytotoxic T cells within the tumor area (280 cells/mm^2^) indicated a robust, pre-existing, but likely suppressed, immune infiltrate. The key to the success of the PD-1 inhibitor may lie not within the tumor cells themselves, but within the stromal compartment. We identified a remarkably high density of CD38+ cells (800 cells/mm^2^) in the stroma. CD38 is a marker associated with T cell activation and a population of plasma cells, suggesting the presence of an active, tertiary lymphoid-like structure (20). Furthermore, the stroma harbored significant populations of other immune cells, including B cells and macrophages. Notably, we identified PD-L1+ macrophages in both the tumor and, more abundantly, in the stroma. This suggests that the PD-1/PD-L1 axis was functionally active in the TME, particularly involving myeloid cells in the stroma, even though tumor cells themselves did not express PD-L1 sufficiently to yield a positive CPS on the initial biopsy (21). By blocking the PD-1 pathway, the therapy likely rejuvenated the functionally exhausted CD8+ T cells and enhanced the anti-tumor activity of other immune cells within this stromal niche, leading to the powerful, coordinated immune attack that eradicated the tumor (22). The immune-rich stromal features observed in the resection specimen may represent a pre-existing receptive microenvironment or may have developed as a consequence of treatment-induced immune activation. Since pre-treatment mIF was not available, the predictive versus reactive nature of these changes cannot be determined. These observations should be considered hypothesis-generating.

In conclusion, this case demonstrates that neoadjuvant chemoimmunotherapy with cisplatin/carboplatin, etoposide, and a PD-1 inhibitor can be a highly effective strategy for downstaging and achieving pCR in unresectable PHSCC, even in a patient with a PD-L1 CPS of 0. The response underscores the limitation of relying on a single biomarker from a small biopsy sample. Comprehensive analysis of the TME, particularly the stromal compartment, is essential for understanding the mechanisms of action. The presence of an immune-rich stroma, featuring activated CD38+ cells and PD-L1+ myeloid cells, may be a critical determinant for the efficacy of immunotherapy in such rare and aggressive malignancies. Further studies are warranted to validate these findings and to explore the role of stromal immune cells as predictive biomarkers for immunotherapy in neuroendocrine carcinomas.

## Limitations

This study has several limitations. First, a pre-treatment tumor microenvironment analysis could not be performed. The diagnostic sample was a core needle biopsy with limited tissue, and despite repeated attempts, multiplex immunofluorescence could not be successfully completed on the remaining material. Therefore, the immune features observed in the resection specimen cannot be directly compared with the pre-treatment microenvironment, and whether they represent a pre-existing receptive state or a treatment-induced change remains unknown. Second, although the patient has remained disease free for 12 months, the follow-up duration is still relatively limited for this aggressive malignancy, and longer observation is required to fully assess the durability of response. Third, no genomic or transcriptomic profiling was performed, limiting our ability to define the molecular background of this rare tumor or to identify predictive biomarkers. Finally, this is a single-case report, and the findings require validation in larger series.

## Conclusion

This case report demonstrates that neoadjuvant chemoimmunotherapy with a platinum-etoposide regimen and a PD-1 inhibitor can be a highly effective strategy for converting unresectable PHSCC into a resectable state and achieving a complete pathological response, even in a patient with a PD-L1 negative (CPS 0) tumor. The remarkable pCR achieved highlights the limitations of relying solely on tumor cell PD-L1 expression as a definitive predictive biomarker for immunotherapy in this rare malignancy.

This case underscores the importance of a multidisciplinary approach and suggests that the composition of the tumor immune microenvironment, particularly the stromal immune infiltrate, may be a more reliable predictor of response than PD-L1 status alone in PHSCC. Further investigation into stromal immune cells as potential biomarkers is warranted to optimize immunotherapeutic strategies for patients with this aggressive and rare cancer.

## Data Availability

The original contributions presented in the study are included in the article/supplementary material. Further inquiries can be directed to the corresponding author.
