# Supplementary material for: Complete pathological response after neoadjuvant chemoimmunotherapy in PD-L1-negative unresectable primary hepatic small cell carcinoma: a case report and tumor microenvironment analysis
Source: Front Immunol. 2026 Sep 11;17:1901608. doi: 10.3389/fimmu.2026.1901608 (PMC13612312; doi:10.3389/fimmu.2026.1901608)
Supplement: Supplementary file 1 [file SupplementaryFile1.docx]

Supplementary materials

Methods

**Multiplex immunohistochemistry and Multichannel imaging**

Deparaffinization of tissue sections was done through xylenes and rehydration through decreasing graded alcohol. AR6 buffer (Akoya Biosciences) was used for antigen retrieval in a microwave oven. Endogenous peroxidase was inactivated by incubation in 3% H2O2 for 10 min. Multiplex immunohistochemistry was performed by several rounds of staining, each including a protein block with 1% BSA followed by primary antibody and corresponding secondary horseradish peroxidase-conjugated antibody against mouse or rabbit immunoglobulins (Akoya Biosciences). The slides were then incubated in different Opal fluorophore (1:100) diluted in 1X Plus Amplification Diluent (Akoya Biosciences). After tyramide signal amplification and covalent linkage of the individual Opal fluorophores (Akoya Biosciences) to the relevant epitope or epitopes, the primary and secondary antibodies were removed via antigen retrieval as previously mentioned and the next cycle of immunostaining was initiated. The sequence of primary antibodies and Opal fluorophores was as follows:

- Panel 1: anti‑CD38 (AB108403, 1:500, Abcam)/Opal 620, anti‑Ki67 (AB16667, 1:200, Abcam)/Opal 520, anti‑CD20 (AB64088, 1:200, Abcam)/Opal 690, anti‑CK (ZM‑0069, 1:1600, ZSGB‑BIO)/Opal 480, and anti‑CD8 (66868‑1‑Ig, 1:80000, Proteintech)/Opal 570.
- Panel 2: anti‑CD66b (ab300122, 1:2000, Abcam)/Opal 620, anti‑PDL1 (13684S, 1:200, Cell Signaling Technology)/Opal 520, anti‑CD68 (GM087629, 1:200, Genetex)/Opal 690, anti‑PD1 (ZM‑0381, 1:200, ZSGB‑BIO)/Opal 570, and anti‑CK (ZM‑0069, 1:1600, ZSGB‑BIO)/Opal 480.

After completion of all cycles, all slides were counterstained with spectral DAPI (Akoya Biosciences) for 10 min and mounted with anti‑fade fluorescence mounting medium (ab104135, Abcam). Multichannel imaging was performed on a PANNORAMIC SCAN II Imaging System (3Dhistech, Hungary) at ×200 magnification.

**Multiplex image analysis**

Image analysis was performed using QuPath v0.4.3 (University of Edinburgh, UK). Tumor and stromal areas were first delineated. DAPI staining was used for whole-slide cell identification, and cells were classified as single-positive, double-positive, or triple-positive based on the staining patterns and subcellular localizations (cytoplasmic, membranous, or nuclear) of each marker, followed by cell quantification. Tissue components were segmented using QuPath’s trainable pixel classifier, which automatically identified specific tissue types based on morphology and texture features after interactive training. The spatial coordinates of individual cells were extracted by the cell segmentation module. For each region of interest, the proportion of each cell phenotype relative to the total cell number within the tumor microenvironment was quantified.
